# Microsatellite Content in 397 Nuclear Exons and Their Flanking Regions in the Fern Family Ophioglossaceae

**DOI:** 10.3390/plants13050713

**Published:** 2024-03-04

**Authors:** Darina Koubínová, GoFlag Consortium, Jason R. Grant

**Affiliations:** Institute of Biology, University of Neuchâtel, Rue Emile Argand 11, 2000 Neuchâtel, Switzerland; jason.grant@unine.ch

**Keywords:** Ophioglossaceae, SSRs, microsatellites, exons, nuclear data, ferns

## Abstract

Microsatellites or SSRs are small tandem repeats that are 1–6 bp long. They are usually highly polymorphic and form important portions of genomes. They have been extensively analyzed in humans, animals and model plants; however, information from non-flowering plants is generally lacking. Here, we examined 29 samples of Ophioglossaceae ferns, mainly from the genera *Botrychium* and *Sceptridium*. We analyzed the SSR distribution, density and composition in almost 400 nuclear exons and their flanking regions. We detected 45 SSRs in exons and 1475 SSRs in the flanking regions. In the exons, only di-, tri- and tetranucleotides were found, and all of them were 12 bp long. The annotation of the exons containing SSRs showed that they were related to various processes, such as metabolism, catalysis, transportation or plant growth. The flanking regions contained SSRs from all categories, with the most numerous being dinucleotides, followed by tetranucleotides. More than one-third of all the SSRs in the flanking regions were 12 bp long. The SSR densities in the exons were very low, ranging from 0 to 0.07 SSRs/kb, while those in the flanking regions ranged from 0.24 to 0.81 SSRs/kb; and those in the combined dataset ranged from 0.2 to 0.81 SSRs/kb. The majority of the detected SSRs in the flanking regions were polymorphic and present at the same loci across two or more samples but differing in the number of repeats. The SSRs detected here may serve as a basis for further population genetic, phylogenetic or evolutionary genetic studies, as well as for further studies focusing on SSRs in the genomes and their roles in adaptation, evolution and diseases.

## 1. Introduction

Eukaryotic genomes are very complex, and despite the enormous number of whole-genome sequencing projects in recent years, we are still not fully aware of the entire genome contents, organization and roles of some parts of the genetic material in evolution, adaptation or in disease development or resistance. DNA itself is composed of protein-coding and non-coding regions. While the purpose of the former is obvious, i.e., encoding for proteins, the latter, which represents the vast majority of the genome (it is estimated to be approximately 99% in humans), was in the early days considered to not have any function. However, it is now becoming more and more evident that certain non-coding regions play important roles in regulatory processes or have some structural functions (despite the roles of the majority of non-coding regions still being unknown). Apart from the coding and non-coding parts, DNA contains non-repetitive and repetitive regions. The latter type usually represents a large fraction and is classified into two categories: tandem repetitive elements (satellite DNA) and interspersed repeats (transposable elements). Satellite DNA includes microsatellites, minisatellites and macrosatellites. Similarly, as in the case of non-coding regions, it was originally thought that these regions do not have any specific functions; however, recent studies reveal potential associations with adaptation, diseases or processes at both the organismal and cellular levels [1,2]. In addition, despite the original estimations, it was later revealed that they are also present in coding regions [3,4,5]. Nevertheless, we are still far from fully understanding the role, evolution or distribution of repetitive elements. In order to tackle this issue, more and more descriptive studies have appeared in recent years; however, most of them are focused on human, animal or model plant genomes.

Microsatellites are the smallest tandem repeats, 1–6 bp in size, and thus, are also called SSRs (simple sequence repeats) or STRs (short tandem repeats). They are widely distributed across the entire prokaryote and eukaryote genomes. They have been detected in both protein-coding and non-coding regions, where they are incorporated into non-repetitive, unique sequences. In animals, it has been shown that approximately 10% of the identified SSRs are located in coding sequences or open reading frames (ORFs) [3]. They are also present in the heterochromatin regions of eukaryotes, where they seem to have a structural role and are important in chromosomal rearrangements [6]. In humans, microsatellite expansions are associated with various diseases [7]. Alterations in microsatellites can affect gene expression and phenotype. They are present in sequences linked to binding functions, catalytic activity, developmental and metabolic processes, or are expressed in membranes and organelles [8]. They are characterized by high polymorphism and a remarkable mutation rate (which surpasses the mutation rate of the point mutations, i.e., insertions, deletions or substitutions) [9]. They have a codominant inheritance, are evenly distributed across the entire genome, and are specific to particular loci (compared to multi-locus markers such as minisatellites). They have long been used in genetic identification, genetic diversity and linkage analyses and fingerprinting. Despite their many advantages for genetic studies, there are also some drawbacks associated with microsatellites. For instance, mutations in the primer annealing site may result in null alleles (existing alleles that are not observed). Additionally, mutations in the flanking regions (or in the microsatellites) may lead to homoplasy (if the scoring is focused only on the fragment length) [10,11]. Additional issues may be related to the primer design and their transferability across related and, especially, more distant species [12,13]. 

In economically important plants, microsatellites are used for molecular assisted breeding. The majority of the descriptive studies are rather focused on models, agriculturally important and flowering (angiosperm) plants, and their plastomes [14,15]. However, investigations of genetic materials other than plastomes and of other plant species are necessary to fully understand the roles, distribution and evolution of microsatellites in plants and in general. Specifically, more investigations of non-flowering and more primitive plants are needed, as well as comparisons of SSRs in organelle and nuclear genomes (which are still mostly missing). Thus, an interesting option might be primitive, eusporangiate ferns, such as the order Ophioglossales, which represent the earliest vascular plants. Moreover, understanding the composition of their genomes, which are often large and complex, is crucial for understanding the evolution of vascular plants. An examination of the microsatellites could contribute to our comprehension of genome evolution, and a descriptive study of the microsatellite distribution in the nuclear genome may provide a basis for future studies focused on their function and role in metabolic, adaptative, structural and other processes.

The order Ophioglossales and its single family Ophioglossaceae are eusporangiate ferns, which together with three other lineages (Psilotales, Equisetales and Marattiales) represent an ancient lineage that differs in many aspects from other, i.e., leptosporangiate, ferns. Currently, there are 112 Ophioglossaceae species recognized and classified into four subfamilies: two monotypic, Mankyuoideae and Helminthostachyoideae, and the more numerous Botrychioideae and Ophioglossoideae [16]. Complete nuclear genomes of Ophioglossaceae are still not available, mainly due to their large sizes and high chromosome numbers, which are among the largest and highest among all organisms (the most up-to-date 1C-values measured for Ophioglossaceae genome sizes range from 6 to 98 pg) [17,18,19]. Descriptive analyses of SSRs are only available for a few plastomes of leptosporangiate ferns [20,21,22]. Recently, SSRs were analyzed in 14 Ophioglossaceae plastomes, which allows us to compare our findings in the nuclear genome and the plastome [23]. A description of the SSR abundance and distribution within genomes and across species is necessary in order to understand SSR functions and distributions in genomes in general and to also potentially reveal the genome evolution in Ophioglossaceae and in ferns and plants. Ophioglossaceae, as a basal lineage, are a crucial milestone in plants’ adaptation to life on land and the clade leading to flowering plants.

Here, we mined the microsatellites from 29 samples of Ophioglossaceae ferns, mainly from the genera *Botrychium* and *Sceptridium*. We analyzed their distribution, density and composition in almost 400 nuclear exons and their flanking regions. The data were compared with those that were previously obtained from Ophioglossaceae plastomes. To our knowledge, this is the first study of microsatellites in the nuclear genome across several species of Ophioglossaceae. The results can serve as a basis for further genetic, phylogenetic, evolutionary and diversity studies.

## 2. Results

### 2.1. SSR Number and Density-Exons

The probe set targeted 451 exons; however, the number of loci obtained for the Ophioglossaceae was lower and ranged from 23 to 412 per species (Table S1). The final TargetOnly.Keep1 dataset contained 397 exon fragments and the Full.Keep1 dataset contained 396 exons + flanking regions (locus L90 excluded, see Details about the datasets in Section 4).

The Keep1 alignment of the exons was 74,353 bp long, with individual sequence lengths ranging from 5522 to 72,435 bp, and contained 11 distinct motifs in 11 distinct exons, i.e., 45 SSRs in total, detected in 24 taxa (Table 1 and Table S2). Most repeats were found in one of the *O. pendula* (5 SSRs) samples, while the other taxa contained one to three SSRs. No SSRs were found in *B. alaskense*, *B. alaskense salchaketense*, *B. lanceolatum*, *B. lunaria* or one of the *O. pendula* species. Except for *B. lunaria*, all species had a high percentage of missing data. The SSR densities were very low and ranged from 0.0138 to 0.07. The lowest density was detected in *S. biternatum* and one of the *B. virginianus* samples; the highest was in one of the *O. pendula* samples.

**Figure 1:**
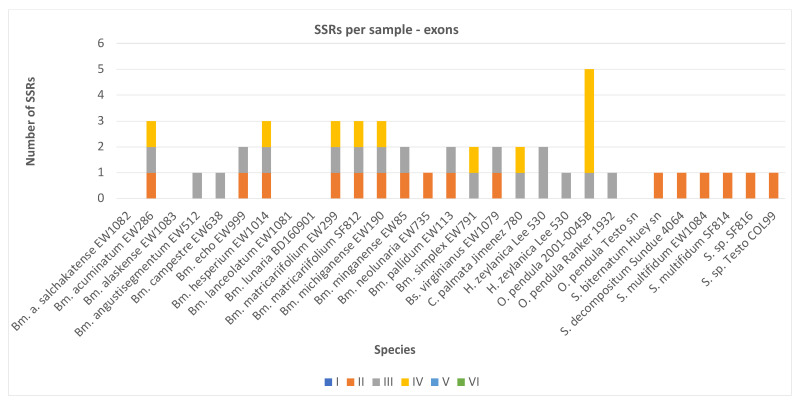
Distribution of mono- to hexanucleotide SSRs in the exons of each Ophioglossaceae sample. Bs.—*Botrypus*; Bm.—*Botrychium*; C.—*Cheiroglossa*; H.—*Helminthostachys*; O.—*Ophioderma*; S.—*Sceptridium*. The colors indicate the number of nucleotides forming the motif (only di- (orange), tri- (grey) and tetranucleotides (yellow) were detected).

The SSR repeats were only di- (16), tri- (18) and tetranucleotides (11) and they repeated mostly three times (six motifs and a total of 11 occurrences), four times (four motifs and 18 occurrences) or six times (one motif and 16 occurrences; Figure 1). All of them were 12 bp long. Eight motifs were unique and three were shared (37 SSRs in total). The SSRs were neither polymorphic nor compound. The most widely shared SSRs were (AG)6 in L221, detected in 16 taxa (i.e., 9 *Botrychium* species, 6 *Sceptridium* and 1 *Botrypus*) and (GCT)4 in L236, present in 15 taxa (i.e., in 12 *Botrychium*, 1 *Helminthostachys*, 1 *Botrypus* and 1 *Cheiroglossa*). (TTCC)3 detected in L415 was shared among six *Botrychium* species. The unique SSRs were detected only in the less-represented species from the Ophioglossoidae (*Ophioderma* and *Cheiroglossa*) and Helminthostachyoideae (*Helminthostachys*) subfamilies.

Three of the detected SSRs were not present in the “NoDups” dataset (two shared occurrences of (GCT)4 in *B. pallidum* and *B. simplex* and one (TTCC)3 in *B. matricariifolium*; see details about the dataset in Section 4).

The annotation of the exons containing SSRs showed that they were related to various processes, such as metabolism, catalysis, transportation, or plant growth (Table S3).

### 2.2. SSR Number and Density—Flanking Regions

The length of the total alignment of the dataset including the exons and flanking regions was 350,929 bp, and the number of nucleotides for individual taxa ranged from 8121 to 306,450 bp, i.e., from 97.69 to 12.67% missing data (Table S2; with the highest portion of missing data in *B. lanceolatum*, *B. alaskense* and the non-Ophioglossoideae species *Ophioderma*,* Helminthostachys* and *Cheiroglossa*). The length of the individual alignments ranged from 159 to 2228 bp (Table S2). There was also a high variability between alignments in terms of the lengths of the obtained fragments per sample.

In this dataset, MISA detected 1520 SSRs in total, i.e., 1475 in flanking regions only (after exclusion of SSRs detected in exons) and in 202 individual flanking region alignments (in total, including the exon regions in 206 alignments that the SSRs were detected in; Table 2 and Table S4). The obtained SSRs were then manually checked and some of them, specifically the compound SSRs, were manually corrected, since, in some cases, when comparing the samples to each other, the SSRs could be interpreted in different ways than those seen in the MISA output (e.g., in cases when a part of the fragment was missing in one sample but was available in other samples, the motif could start at different codon positions, i.e., AAG, AGA, or GAA, and the comparison of several samples indicated one as mostly probable and shared, or the MISA output was too complicated, as it attempted to indicate the sharing of some of the nucleotides between the individual SSR units in the compound SSRs, while the observed situation could be interpreted more simply, e.g., (GTAT)3(GT)12 instead of the (GTAT)2(GTA < T > G)(TG)12 output by MISA; Table S5).

SSRs were detected in all samples; however, one of the samples (*B. alaskense*) only contained one SSR. This is likely a consequence of the amount of missing data in this sample—almost 98%. The remaining 28 samples each contained multiple SSRs (3−103). The SSR densities ranged from 0.20 to 0.81. The highest densities were detected in *B. lanceolatum* and *B. alaskense salchaketense*, whereas the lowest densities were found in *H. zeylanica* and one of the *O. pendula* samples. The SSR density in *B. alaskense*, the sample with the most missing data, was 0.38, i.e., not the lowest. The observed density in the combined dataset ranged from 0.2 to 0.81.

**Figure 2:**
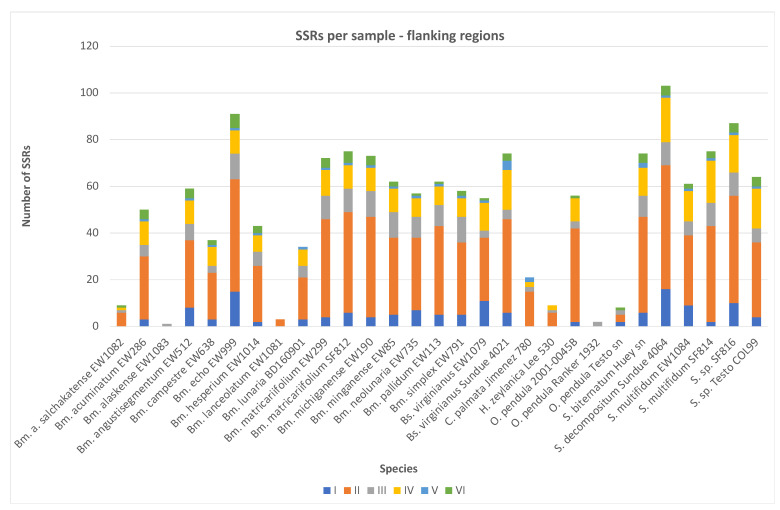
Distribution of mono- to hexanucleotide SSRs in the flanking regions in each of the Ophioglossaceae samples. Bm.—*Botrychium*; Bs.—*Botrypus*; C.—*Cheiroglossa*; H.—*Helmintostachys*; O.—*Ophioderma*; S.—*Sceptridium*. The colors indicate the number of nucleotides forming the motif, i.e., mono- (dark blue) to hexanucleotides (green).

A total of 138 SSRs were mononucleotide, 810 were dinucleotide, 178 were trinucleotide, 256 were tetranucleotide, 27 were pentanucleotide, and 66 were hexanucleotide repeats (Figure 2). The highest number of repeats was (AG)34, followed by (GA)29 and (C)29. The longest repeat was (AG)34 (68 bp), followed by (GA)29 (58 bp) and (GA)28, (AG)28 and (TC)28 (all 56 bp). The highest frequency of occurrence of a repeat was AG, which was present in the dataset in various numbers of repeats 253 times, followed by GA (140 times) and TC (98 times). When considering the complementarity of the repeats, the (AG)6/(CT)6 motif, with 117 occurrences, and (AG)11/(CT)11, with 95 occurrences, were the most frequent ones. AG/CT in various numbers of repeats was present 594 times in total, and AC/GT occurred 213 times. The tetra-, penta- and hexanucleotides were present only in forms that were repeated up to nine times (AAAAGA—nine times in *Botrypus virginianus*); the majority of them consisted, however, of three to four repeats.

**Figure 3:**
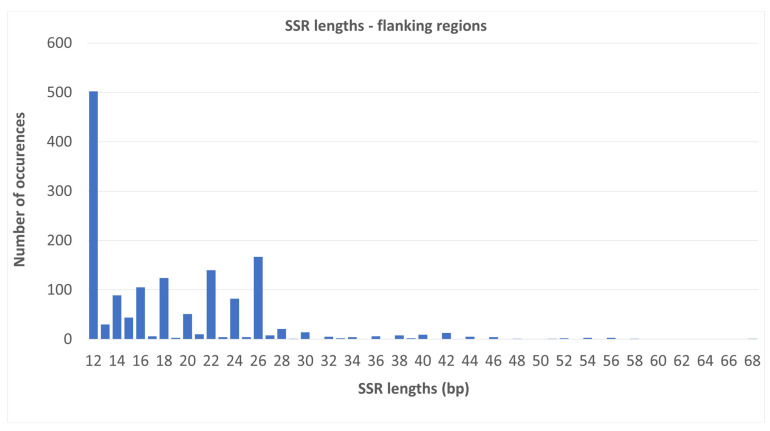
Lengths of the SSR motifs in the flanking regions. The *x*-axis indicates the length of the SSR repeats (in bp) and the *y*-axis indicates the number of occurrences of repeats in the flanking regions.

More than one-third of the SSRs (502, 34.03%) were 12 bp long, with other frequently occurring lengths being 26, 22, 18 and 16 bp (167, 140, 124, and 105 occurrences, respectively; Figure 3).

### 2.3. Unique, Shared and Polymorphic SSRs

All three categories (unique, polymorphic and shared) were detected in almost all species. However, *B. alaskense* only contained one shared SSR, *P. lanceolatum* had three polymorphic SSRs and *B. alaskense salchaketense* and *B. acuminatum* only contained polymorphic and shared SSRs. *O. pendula* and *C. palmata* contained mainly unique SSRs and a few polymorphic ones. Some SSRs were unique but were aligned at the same loci as the polymorphic ones: three SSRs in three *Botrychium* species, or, in one case in *Botrypus* (CA)9 and in *Botrychium*, (TA)9 was detected at the same locus.

There were 176 unique SSRs in 128 loci in 25 samples and in all categories (42 in mononucleotides, 73 dinucleotides, 16 trinucleotides, 32 tetranucleotides, 6 pentanucleotides and 7 hexanucleotides); the remaining 1299 occurred across samples either in a polymorphic or in the same shared form. The most unique SSRs were detected in one of the *O. pendula* species (48), while in others, the number of unique SSRs ranged from 1 to 28.

Out of the 1299 SSRs, 900 in 81 loci in 27 samples were polymorphic: 699 SSRs in 53 loci in 27 samples were polymorphic across genera, 199 SSRs in 35 loci were shared among 21 samples where the polymorphism was detected within the same genus, and 2 SSRs were detected among different samples within the same species, *B. matricariifolium*. However, this “polymorphism” is apparently only a result of an incomplete SSR due to its occurrence at the incomplete beginning of the loci. We consider it here as polymorphic, but it is highly probable that it is a shared SSR. No pentanucleotides were among the polymorphic SSRs across the genera and species, 83 were mononucleotides (55 shared across genera, 28 within genera), 691 were dinucleotides (584 shared across genera, 106 within genera, 2 within species), 44 were trinucleotides (22 shared across genera, 22 within genera), 56 were tetranucleotides (27 shared across genera, 29 within genera) and 25 were hexanucleotides (11 shared across genera, 14 within genera). The length of the polymorphic SSRs was 12 to 68 bp.

The remaining 399 SSRs in 65 loci and 24 samples were shared. A total of 153 SSRs at 15 loci and in 24 samples were shared across genera, 228 at 43 loci in 20 species within genera and 18 in 8 loci between samples of the same two species, *Botrypus virginianus* and *Botrychium matricariifolium*. SSRs were detected in all categories: 13 mononucleotides (shared within genera), 45 dinucleotides (19 shared across genera, 20 shared within genera, 6 within species), 118 trinucleotides (46 shared across genera, 72 shared within genera), 168 tetranucleotides (88 shared across genera, 70 shared within genera, 10 shared within species), 21 pentanucleotides (19 shared within genera, 2 shared within species) and 34 hexanucleotides (shared within genera). There were no mono-, penta- or hexanucleotides among the SSRs shared across genera, and all of them were 12 bp long. All categories were represented among the SSRs shared within a genus, and di-, tetra- and pentanucleotides were represented among the SSRs shared within species; however, there were also lengths other than 12 bp in both groups, i.e., up to 46 bp in those shared within genera and up to 28 bp in those shared within species. The SSRs shared within genera were shared among 3–19 samples, and there were 1 to 32 SSRs per sample. The most commonly shared SSRs were (GCAT)3 in L26, which was detected in 19 samples; (TTTG)3 in L53 in 18 samples; (AG)6 in L220 and (GAGG)3 in L412 in 15 samples; (GAT)4 in L41 in 14 samples; and (GCT)4 in L237 in 14 samples.

The SSRs shared within genera were shared among 2–13 samples, and there were 2 to 22 SSRs per sample. The most commonly shared SSRs were (ATG)4 in L275 and L276, which was detected in 13 *Botrychium* samples; (TCTCC)3 in 13 *Botrychium* samples; (GGGT)3 in L407 in 10 *Botrychium* samples; (TTTCTT)3 in L410 in 10 *Botrychium* samples; (TGC)4 in L102 in 9 *Botrychium* samples; and (TTTA)3 in L138 in 9 *Botrychium* samples.

### 2.4. Compound Form

A total of 61 SSRs (47 distinct motifs) in 25 exon + flanking region fragment alignments were present in a compound form: 48 in the form where the repeats were next to each other, and the remaining 13 were detected as overlapping (we corrected these manually to simpler, “classic” compound forms, as mentioned above). Nine SSRs (seven distinct motifs) were composed of three SSRs, while the rest (52 SSRs in total and 40 distinct) were composed of two. The longest composed motif was 86 bp long, while the shortest was 24 bp (10 to 56 bp for the simple SSRs). In general, slightly more SSRs were found among the 36, 24 and 54 bp SSRs. The highest number of repeats of simple SSRs was 28 for a GA dinucleotide. The majority of the SSRs were composed of two dinucleotide repeats or a combination of di- and tetranucleotide repeats; however, motifs composed of two hexanucleotide repeats were also detected. In total, the compound SSRs consisted of 12 mono-, 84 di-, 10 tri-, 10 tetra- and 15 hexanucleotide SSRs (131 in total). No pentanucleotide motif was detected among the compound SSRs. One fragment (L256, a middle-sized one, 743 bp in length) contained two distinct compound SSRs. There was no compound SSR in *Cheiroglossa*.

A total of 13 SSRs were unique, i.e., only detected in one specimen; 36 SSRs (more than a half of the total number) were polymorphic, and 12 were shared. Neither polymorphic nor shared SSRs were detected in *Ophioderma* or *Helminthostachys*, and no shared SSRs were detected in genera other than *Sceptridium*, i.e., polymorphic SSRs occurred only within or between at least two (or all three) closely related genera from the subfamily *Botrychioideae* (*Botrychium*, *Sceptridium* and *Botrypus*) and the shared ones only among *Sceptridium* species or within two pairs of samples representing the same species.

In 24 cases (more than one-third of the total number of SSRs detected), the polymorphism also included some variation in the motifs, e.g., only one part of the motif was similar (e.g., (CTCC)3(C)23 vs. (C)18(TC)6 or (TC)9(C)12 polymorphisms among three samples), or there were, for example, some point mutations which caused a shift in the motif (e.g., (AAG)*n* in one sample vs. (AGA)*n* in another, which is sometimes caused by the fact that they were missing data at the beginning, as the fragment started with the SSR) or some motifs at the same loci were composed of two SSRs in some samples and of three in other samples. In one case, one of the motifs forming the compound SSR was tetranucleotide, while it was trinucleotide in another SSR (e.g., (AAGA)*n* in *Botrychium* but (AAG)*n* in *Botrypus*), or there was a combination of some of the above-mentioned cases (e.g., (TC)8(TA)9 vs. (CT)9(CA)9).

A potential polymorphism between the two samples of *B. matricariifolium* was detected; however, a manual inspection revealed that the discrepancy was caused mainly by a premature end of one of the fragments, causing the second repeat to be incomplete. We marked this SSR as polymorphic; however, it is highly probable that it is the same compound SSR present in both samples. One case of polymorphism within two species of *Botrychium* (in one of the species the compound motif was composed of two motifs and in the other species, it was composed of three motifs) may be again caused by the occurrence at the beginning of the fragment, i.e., missing data. A total of 32 SSRs (i.e., approximately half of the compound SSRs detected) present in 7 fragments were polymorphic across species and genera. The most commonly occurring compound polymorphic SSR (TG)*n*/AG)*n* was present in 10 species and three genera (*Botrychium*, *Botrypus*, *Sceptridium*) in L379 (in this case, neither GT/TG nor AG/GA polymorphism was observed, i.e., the TG and AG motifs were in the same form in all samples).

In some cases, compound SSRs were only detected in two samples from distinct genera but not in the other species from the same genus. In some cases, it was a point mutation or another alteration of the motifs; however, in some cases, it was also due to missing data. In five cases, the SSRs were polymorphic among the samples from two genera, and in one case, they were polymorphic between three genera.

Four SSRs were detected as shared among samples of the same species (two from *Botrypus virginianus* and two from *Botrychium matricariifolium*). Ten other SSRs (two distinct motifs) were shared within different species of *Sceptridium*: one of the motifs was composed of two dinucleotide repeats (shared by two species; total length of 50 bp) and one was a 2 hexanucleotide species (shared by five species if we consider the two *Sceptridium* sp. as two distinct species; total length of 36 bp).

The compound SSRs were also considered single SSRs; however, (T)10 was excluded, as it was below our threshold.

### 2.5. Primer Design

The default settings enabled us to design primers for 469 out of the 1520 SSRs (Table S6). In some cases, it was not possible to design primers, as the SSRs were at the ends of the fragments. 

## 3. Discussion

We analyzed the microsatellites or SSRs distributed in almost 400 nuclear exons in 29 samples of Ophioglossaceae ferns.

The majority of the SSRs were polymorphic or shared. As polymorphism occurs through a reduction or increase in the number of SSRs, it is possible that they were originally also shared, i.e., similar and the differences in the number of repeats occurred later. In a previous study where we examined the SSRs in plastomes of 14 Ophioglossaceae ferns, we found out that most of the SSRs were unique [23]. However, this discrepancy between the observations here and those in the previous study is at least partially caused by the fact that here, we analyzed only certain species, mainly from one subfamily and two genera, while in the previous study, we analyzed species across the whole Ophioglossaceae family, and there may be also a bias since the whole plastome sequence was analyzed, while here, we only focused on exons and adjacent flanking regions where the density of SSRs can differ from the rest of the nuclear genome. Nevertheless, if only the plastomes of the *Sceptridium* and *Botrychium* samples were compared, they still contained a high portion of unique SSRs. However, there were also some SSRs that were only shared among some of the species of the respective genera and not all of them. A more in-depth investigation is thus necessary, especially regarding the plastomes of more species in order to correctly determine the prevalence of unique shared and polymorphic data in the nuclear genomes and plastomes of Ophioglossaceae.

The exon regions contained only di-, tri- and tetranucleotides, while the flanking regions contained mono- to hexanucleotides, with the most represented being dinucleotides, followed by tetranucleotides. In individual species, dinucleotides were the most numerous (except some species whose samples contained much missing data and only a few SSRs); however, in five samples (with a sufficient amount of data), trinucleotides were more common than tetranucleotides, and in one case, they were equally represented. In the plastomes [23], mononucleotides were the most prevalent SSRs. This is in concordance with previous studies, where no fixed pattern was detected, and different organisms, organismal groups or genomes had specific predominant SSRs. However, a comprehensive study of all complete gene-coding sequences in more than one hundred plants revealed trinucleotides as dominant in all groups (including higher and lower plants) [5].

Previously, a discrepancy between the prevalence of the type of motif in chloroplast and mitochondrial genomes was observed [15]. On the other hand, the *H. zeylanica* in our study showed low SSR densities in both the plastome and nuclear datasets. Furthermore, mononucleotides were found to be predominant in Ophioglossaceae and other fern plastomes. Further investigations of organelle and nuclear genomes are thus needed to find any potential similarities in patterns.

The SSR densities in exons were very low, ranging from 0 to 0.07 SSRs/kb; 0.24 to 0.81 SSRs/kb in flanking regions; and from 0.2 to 0.81 SSRs/kb in the combined dataset. The overall (and the flanking region) density was thus slightly higher than the overall density observed in the plastomes (i.e., 0.14–0.29 SSRs/kb; [23]). A recent study [24] detected a lower density in bamboo nuclear genomes (0.09 to 0.16 SSRs/kb), despite the fact that in this study, hepta- to decanucleotides were also considered microsatellites and, thus, the mono- to hexanucleotide density would be substantially lower. Similar low densities in exons were detected in the genes of selected vascular non-flowering plants (0.01–0.09 SSRs/kb, with hepta- to enneanucleotides considered SSRs, i.e., the mono- to hexanucleotide densities would be lower; [5]). Similarly, the number of genes with SSRs (2.77%) is in the range found in a previous study (1.23–9.78%; [5]).

From the data, lower numbers of repeats appear to occur as more motifs but were less frequent than higher repeat numbers, which were highly shared. The SSRs detected in the exons were unique or shared. No polymorphic or compound SSRs were detected. All the motifs found in exons based on the MISA settings were 12 bp long only. In the Ophioglossaceae whole plastome data [23], however, the lengths of the SSRs detected in exons were between 12 and 44 bp. In this study, in the flanking regions in the nuclear data, slightly more than one-third of the SSRs were also 12 bp long. The most abundant were trinucleotides; however, this prevalence is biased by the low number of detected SSRs, missing data, and the fact that many of them were shared and abundant in one exon. Trimers were previously detected as the most abundant motifs in *Picea abies* for longer SSRs (>20 bp) and were equally as abundant as hexamers in *P. taeda* [25].

It was previously suggested that SSRs in plants may play a role in fast adaptation to environmental changes [26]. In general, Ophioglossaceae and ferns are known to have large genomes. It was shown that the genome size is correlated with the habitat type and, thus, with adaptation [27]. On the other hand, another study showed that the SSR number was higher in primitive plants such as the *Micromonas* algae (0.55 SSRs/kb), while it was relatively low in primitive vascular plants such as lycophytes and ferns [5], and a similar observation was detected here. The authors of the study [5] posit that this higher density may be related to the extreme conditions that primitive plants need to survive. From this perspective, currently, it does not seem that SSRs would play an important role in fern or vascular plant evolution, as their number is low. However, some SSRs were detected in genes related to metabolism, catalysis, transportation and plant growth. This may still indicate that SSRs might have played an important role in the adaptation of Ophioglossaceae ferns. Further studies, specifically on the SSRs in the transcriptome, are thus needed to fully address this question.

The shared and polymorphic SSRs detected here may serve as a basis for further population genetics and similar studies using microsatellites, as well as for further studies focusing on SSRs in the genomes and their roles in adaptation, evolution and diseases.

## 4. Materials and Methods

### 4.1. Samples

Sequences of approximately 400 exons and their corresponding flanking intron regions from twenty-nine samples from the fern family Ophioglossaceae were analyzed (Table S1). The data were provided by the GoFlag project, which is focused on understanding the evolution of flagellate plants through a sequence capture approach with probes targeting nuclear loci across all flagellate plants (i.e., bryophytes, lycophytes, ferns and gymnosperms; [28]). The dataset included samples assigned to the genera *Botrychium* (16 samples; 14 distinct species), *Botrypus* (2 samples; 1 species), *Cheiroglossa* (1 sample; 1 species), *Helminthostachys* (1 sample, 1 species), *Ophioderma* (3 samples; 1 species) and *Sceptridium* (6 samples; 3 distinct species + 2 samples only assigned to a genus).

The probe set for the target enrichment sequencing was designed for 451 exons of the 248 single- or low-copy nuclear genes shared across all flagellate land plants (i.e., from mosses to gymnosperms) and non-flagellate outgroups. The probes were designed to cover conserved exons among a set of more than 400 single-copy (or low-copy) nuclear genes identified by the 1KP initiative [29]. Available genome sequences from land plants were used to identify exons that were at least 120 bp in length. Exons and splice sites conserved across a large diversity of land plants were used to design the probe kit. For the detailed protocol of the probe design, taxon selection, DNA extraction, library construction, target enrichment, sequencing and bioinformatic analyses, as well as additional information about our input data, see the original publication [28].

Here, we used two datasets, one containing the exons only (referred to as “TargetOnly” in the original publication) and exons with the flanking regions (“Full” in the original publication). We analyzed the “Keep1” version (i.e., in cases where there was more than one sequence per sample retained after processing the pipeline, only a single sequence containing the highest number of nucleotides was kept for the final output; see original publication and Supplementary data for details). We refer to the loci using the same system as in the original publication, using the letter L and corresponding number (e.g., L1). In the case of the “TargetOnly” dataset, we also compared the results from the “Full” file with the “NoDups” file (i.e., in cases where there was more than one sequence per sample retained after processing the pipeline, no sequence was used for the output) in order to see potential differences). The raw sequence reads are available in the Sequencing Read Archive (SRA; https://www.ncbi.nlm.nih.gov/sra/, accessed on 27 August 2023) with the following Accessions: SRR27918008-SRR27918036 (BioSamples: SAMN39888241-SAMN39888269). Five of the samples within this submission are not public as they were submitted to the SRA and BioSample databases previously [28], see all the Accessions in Table S1. 

### 4.2. SSR Mining and Primer Design

Perfect (formed by pure repeats of a specific motif) and compound (formed by two or more repeats) SSRs were mined using MISA v. 2.1 (MIcroSAtellite identification tool; command-line version; ref. [30] using the following settings for minimal repeat sizes: 12 for mononucleotides, 6 for dinucleotides, 4 for trinucleotides and 3 for penta-, tetra- and hexanucleotides). We considered two or more SSRs to be compound only if they were adjacent to each other without any interspersed nucleotides. The analyses were performed on each exon or each exon + flanking region alignment separately; the gaps were removed from the alignments before performing the analyses (as MISA would not consider microsatellites with introduced gaps). In the exon + flanking region dataset, we mined the whole dataset and then excluded the data occurring in exons (i.e., the same as that detected in the “exon only” dataset).

The mined SSRs were categorized as unique (not present in another species), polymorphic (i.e., the repeat shows length variations in different species) or shared (present in more than one species). The latter two were further classified based on their occurrence among the samples of the same species, within the same genus only, or across more than one genus. Repeats forming the compound SSRs were also split and analyzed separately as single SSRs. SSR density was calculated as the number of SSRs per kb (i.e., (total number of SSRs/sequence length) × 1000).

The exon fragments containing SSRs were annotated with blastx [31] and OMA browser [32].

We designed primers for the SSR regions with the p3_in_v2.pl and p3_out_v2.pl perl scripts provided from the MISA website and Primer3 [33]. We used the default settings provided in the perl scripts. The original probe set used to obtain the whole fragments is described in the original publication [28].

## Figures and Tables

**Table 1:** Number of SSRs present in each taxon in each category ((I) mono-, (II) di-, (III) tri-, (IV) tetra-, (V) penta- and (VI) hexanucleotides) based on the length of the repeat motif, the total number of simple SSRs detected, the sequence length (i.e., number of nucleotides in bp) and the SSR density (calculated as the number of SSRs per kbp, i.e., (total number of SSRs/sequence length in bp) × 1000).

Species	Voucher	Sequencing ID	Repeat Category-Exons						Total Number	No. of Nucleotides in Exons (bp)	SSR Density
			I	II	III	IV	V	VI			
*Botrychium acuminatum*	EW286	P015 WG11	0	1	1	1	0	0	3	66,866	0.0449
*Botrychium alaskense*	EW1083	P03 WD06	0	0	0	0	0	0	0	5522	0
*Botrychium* *alaskense salchakatense*	EW1082	P03 WC06	0	0	0	0	0	0	0	21,221	0
*Botrychium* *angustisegmentum*	EW512	P015 WA12	0	0	1	0	0	0	1	68,226	0.0147
*Botrychium campestre*	EW638	P015 WB12	0	0	1	0	0	0	1	66,839	0.015
*Botrychium echo*	EW999	P015 WE12	0	1	1	0	0	0	2	71,166	0.0281
*Botrychium hesperium*	EW1014	P015 WF12	0	1	1	1	0	0	3	67,467	0.0445
*Botrychium lanceolatum*	EW1081	P03 WB06	0	0	0	0	0	0	0	6905	0
*Botrychium lunaria*	BD160901	P015 WG12	0	0	0	0	0	0	0	60,524	0
*Botrychium* *matricariifolium*	EW299	P015 WH11	0	1	1	1	0	0	3	69,113	0.0434
*Botrychium* *matricariifolium*	SF812	P028 WD08	0	1	1	1	0	0	3	70,879	0.0423
*Botrychium michiganense*	EW190	P015 WF11	0	1	1	1	0	0	3	70,189	0.0427
*Botrychium minganense*	EW85	P015 WD11	0	1	1	0	0	0	2	70,243	0.0285
*Botrychium neolunaria*	EW735	P015 WC12	0	1	0	0	0	0	1	70,226	0.0142
*Botrychium pallidum*	EW113	P015 WE11	0	1	1	0	0	0	2	70,056	0.0285
*Botrychium simplex*	EW791	P015 WD12	0	0	1	1	0	0	2	69,654	0.0287
*Botrypus virginianus*	Sundue 4021	P055 WB10	0	0	1	0	0	0	1	72,233	0.0138
*Botrypus virginianus*	EW1079	P03 WA06	0	1	1	0	0	0	2	72,435	0.0276
*Cheiroglossa palmata*	Jimenez 780	P071 WF12	0	0	1	1	0	0	2	36,029	0.0555
*Helminthostachys* *zeylanica*	Lee 530	P069 WF05	0	0	2	0	0	0	2	52,149	0.0384
*Ophioderma pendula*	2001-0045B	P016 WH10	0	0	1	4	0	0	5	70,580	0.0708
*Ophioderma pendula*	Ranker 1932	P067 WA10	0	0	1	0	0	0	1	17,091	0.0585
*Ophioderma pendula*	Testo sn	P069 WH03	0	0	0	0	0	0	0	25,543	0
*Sceptridium biternatum*	Huey sn	P072 WC08	0	1	0	0	0	0	1	72,420	0.0138
*Sceptridium* *decompositum*	Sundue 4064	P063 WA11	0	1	0	0	0	0	1	71,323	0.014
*Sceptridium multifidum*	SF814	P028 WE08	0	1	0	0	0	0	1	70,552	0.0142
*Sceptridium multifidum*	EW1084	P03 WE06	0	1	0	0	0	0	1	71,229	0.014
*Sceptridium* sp.	SF816	P028 WG08	0	1	0	0	0	0	1	71,359	0.014
*Sceptridium* sp.	Testo COL99	P058 WH09	0	1	0	0	0	0	1	69,912	0.0143
Total number of SSRs per category			0	16	18	11	0	0	45		

**Table 2:** Number of SSRs present in each taxon in the flanking regions in each category ((I) mono-, (II) di-, (III) tri-, (IV) tetra-, (V) penta- and (VI) hexanucleotides) based on the length of the repeat motif, the total number of simple SSRs detected, the number of compound SSRs, the sequence length (i.e., number of nucleotides in bp), and the SSR density (calculated as the number of SSRs per kbp, i.e., (total number of SSRs/sequence length in bp) × 1000).

Species	Voucher	Sequencing ID	Repeat Category-Flanking Region						Total Number	Compound	No. of Nucleotides	SSR Density
			I	II	III	IV	V	VI				
*Botrychium acuminatum*	EW286	P015 WG11	3	27	5	10	1	4	50	1	124,140	0.4028
*Botrychium alaskense*	EW1083	P03 WD06	0	0	1	0	0	0	1	0	2608	0.3834
*Botrychium alaskense* *salchakatense*	EW1082	P03 WC06	0	6	1	1	0	1	9	0	12,969	0.694
*Botrychium* *angustisegmentum*	EW512	P015 WA12	8	29	7	10	1	4	59	0	148,071	0.3985
*Botrychium campestre*	EW638	P015 WB12	3	20	3	8	1	2	37	0	106,754	0.3466
*Botrychium echo*	EW999	P015 WE12	15	48	11	10	1	6	91	6	198,825	0.4577
*Botrychium hesperium*	EW1014	P015 WF12	2	24	6	7	1	3	43	3	134,642	0.3194
*Botrychium lanceolatum*	EW1081	P03 WB06	0	3	0	0	0	0	3	0	3714	0.8078
*Botrychium lunaria*	BD160901	P015 WG12	3	18	5	7	1	0	34	1	106,522	0.3192
*Botrychium matricariifolium*	EW299	P015 WH11	4	42	10	11	1	4	72	3	164,355	0.4381
*Botrychium matricariifolium*	SF812	P028 WD08	6	43	10	10	1	5	75	3	202,143	0.371
*Botrychium michiganense*	EW190	P015 WF11	4	43	11	10	1	4	73	4	172,060	0.4243
*Botrychium minganense*	EW85	P015 WD11	5	33	11	10	1	2	62	0	166,287	0.3728
*Botrychium neolunaria*	EW735	P015 WC12	7	31	9	8	1	1	57	2	154,150	0.3698
*Botrychium pallidum*	EW113	P015 WE11	5	38	9	8	1	1	62	0	177,548	0.3492
*Botrychium simplex*	EW791	P015 WD12	5	31	11	8	1	2	58	1	135,075	0.4294
*Botrypus virginianus*	EW1079	P03 WA06	11	27	3	12	1	1	55	1	152,100	0.3616
*Botrypus virginianus*	Sundue 4021	P055 WB10	6	40	4	17	4	3	74	7	198,398	0.373
*Cheiroglossa palmata*	Jimenez 780	P071 WF12	0	15	2	2	2	0	21	0	32,181	0.6526
*Helminthostachys zeylanica*	Lee 530	P069 WF05	0	6	1	2	0	0	9	1	46,126	0.1951
*Ophioderma pendula*	2001-0045B	P016 WH10	2	40	3	10	0	1	56	2	112,807	0.4964
*Ophioderma pendula*	Ranker 1932	P067 WA10	0	0	2	0	0	0	2	0	6704	0.2983
*Ophioderma pendula*	Testo sn	P069 WH03	2	3	2	0	0	1	8	1	20,763	0.3853
*Sceptridium biternatum*	Huey sn	P072 WC08	6	41	9	12	2	4	74	2	193,043	0.3833
*Sceptridium decompositum*	Sundue 4064	P063 WA11	16	53	10	19	1	4	103	7	237,178	0.4343
*Sceptridium multifidum*	EW1084	P03 WE06	9	30	6	13	1	2	61	4	146,786	0.4156
*Sceptridium multifidum*	SF814	P028 WE08	2	41	10	18	1	3	75	5	205,941	0.3642
*Sceptridium* sp.	SF816	P028 WG08	10	46	10	16	1	4	87	5	213,200	0.4081
*Sceptridium* sp.	Testo COL99	P058 WH09	4	32	6	17	1	4	64	2	151,207	0.4233
Total number of SSRs per category			138	810	178	256	27	66	1475	61		

## Data Availability

The samples used in this study are available in the Sequencing Read Archive (SRA; https://www.ncbi.nlm.nih.gov/sra/, accessed on 27 August 2023) with the Accessions specified in Table S1.

## Supplementary Materials

The following supporting information can be downloaded at https://www.mdpi.com/article/10.3390/plants13050713/s1, Table S1: List of Ophioglossaceae species used. The column Loci indicates the number of exon loci obtained by target enrichment sequencing. The columns SRA Accession and BioSample indicate the associated Accessions in the Sequencing Read Archive (SRA) and BioSample databases at NCBI (https://www.ncbi.nlm.nih.gov/, accessed on 27 August 2023). Five samples were already submitted to the databases previously [28], in such cases the current submission is not public (supressed) which is indicated by brackets; Table S2: List of Ophioglossaceae species used with missing data in the alignment, % of missing data, number of successfully sequenced nucleotides, number of nucleotides detected, and the SSR density. The color scale ranges from green (highest values) to red (lowest values); Table S3: SSRs detected in exons. The colors of the cells in the SSR column indicate unique (red), shared (light green), and shared only within genus SSRs (dark green); Table S4: SSRs detected in the flanking regions. The colors of the cells in the SSR column indicate unique (red), shared (light green), shared only within genus (dark green), shared only within a species (light orange), polymorphic (turquoise), polymorphic only within genus (orange), and polymorphic only within a species (dark blue); Table S5: SSRs detected in a compound form. The colors of the cells in the SSR column indicate unique (red), shared only within genus (dark green), shared only within a species (light orange), polymorphic (turquoise), polymorphic only within genus (orange), and polymorphic only within a species (dark blue); Table S6: Primers designed using Primer3 for the loci flanking the SSRs.
